# Efficacy and Safety of Subacromial Corticosteroid Injection in Type 2 Diabetic Patients

**DOI:** 10.1155/2018/9279343

**Published:** 2018-09-20

**Authors:** Davide Blonna, Davide Edoardo Bonasia, Lorenzo Mattei, Enrico Bellato, Valentina Greco, Roberto Rossi

**Affiliations:** ^1^AO Ordine Mauriziano Umberto I, Department of Orthopedics and Traumatology, Largo Turati 62, 10128 Turin, Italy; ^2^University of Turin, Via Po 8, 10100 Turin, Italy; ^3^San Luigi Gonzaga of Orbassano, Department of Orthopedics and Traumatology, Regione Gonzole 10, 100443 Orbassano, Turin, Italy

## Abstract

**Purpose:**

In type 2 diabetic patients affected by chronic shoulder pain, subacromial injection with corticosteroid could be an effective treatment. The aim of this study was to measure the risk-benefit ratio of this treatment.

**Methods:**

Twenty patients with well-controlled diabetes were included in a prospective study. In a first preinjection phase, patients were asked to measure glycemia for 7 days, before breakfast and dinner, and then 2 hours after lunch and dinner. Baseline data including Constant Score (CS), Subjective Shoulder Value (SSV), and Numerical Rating Scale (NRS) for pain were collected. Patients were treated with subacromial injection with 40 mg of Methylprednisolone Acetate and 2 ml of Lidocaine. At discharge, patients were asked to remeasure glycemia for the following week.

**Results:**

The overall pain improved and patients graded their shoulder as “greatly improved”. At 30-day follow-up, the SSV and the CS improved, considering pain but not ROM. The average daily glycemia was 136 mg/dl before injection, 161 mg/dl the day of the injection (p<0.001), and 170 mg/dl one day after injection (p<0.001). Glycemia was not statistically different 3 days after injection.

**Conclusion:**

Subacromial injection is an effective short-term treatment in type 2 diabetic patients affected by shoulder pain, but a closed follow-up is recommended in all these patients. This trial is registered with NCT03652480. The Protocol ID is SHOULDERDM2013.

## 1. Introduction

Among the conservative strategies to treat shoulder pain, the use of corticosteroids in the subacromial space has been shown to be particularly effective and safe. One of the main limitations for the use of the corticosteroid injections remains the potential effect on blood glucose levels especially in diabetic patients. On the other hand, diabetic patients with noncontrolled shoulder pain could have a major benefit from a corticosteroid injection due to the increased risk of surgical complication: they are often elderly patients, affected by other severe comorbidities, and are strongly reticent to undergo any type of surgery. Nevertheless, they often ask for a rapid, even if temporary, pain relief solution and injection with corticosteroids could be an effective option.

The use of corticosteroids in joints, other than the shoulder, has been widely documented in nondiabetic and in diabetic patients, proving to be safe and effective [1–5]. However, only two studies focused on the painful shoulder and both of them included patients affected by adhesive capsulitis treated with intra-articular corticosteroid injection: the risk of hyperglycemia after injection seemed to be negligible [6, 7].

In our clinical practice, we have observed that (1) diabetic patients with subacromial pain respond very well to subacromial corticosteroid injections in terms of pain relief, but the effect on range of motion (ROM) seems to be unpredictable; (2) some patients report high levels of blood glucose after subacromial injections, an increase that seems to be different from what has been reported after intra-articular injections. The goal of our study was to document the effect of subacromial corticosteroid injections on blood sugar levels and short-term clinical outcomes in patients affected by type-2 diabetes, not suitable for surgery and nonresponsive to other conservative treatments.

## 2. Methods

**Figure 1:**
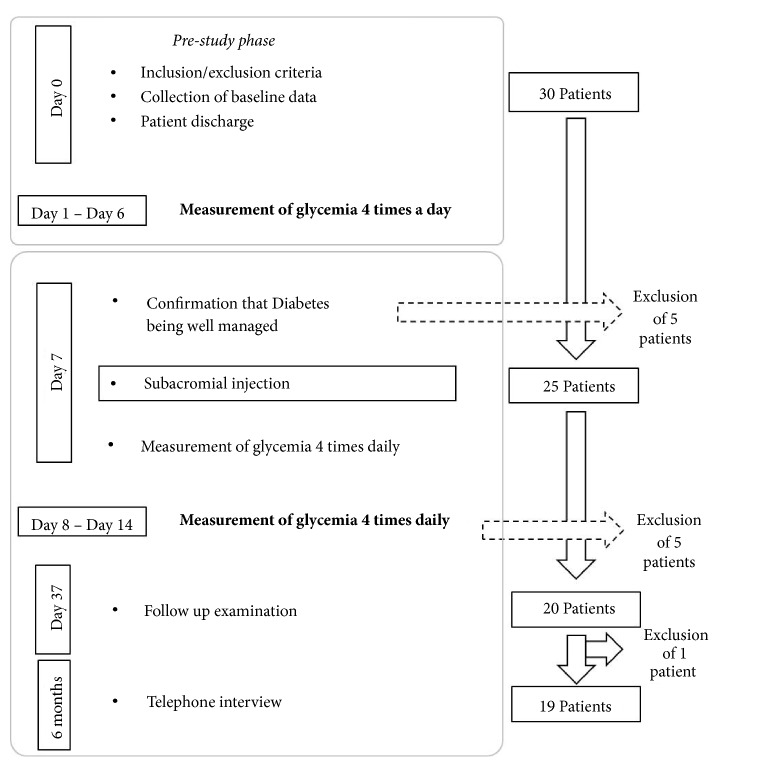
Timeline of the study protocol.

From 2013 to 2014, thirty consecutive type 2 diabetic patients were initially included in this analytic prospective study (level of evidence III). Patients examined in our outpatient department, complaining of chronic shoulder pain resistant to at least 6 months of conservative therapy (NSAIDs, painkillers, and physiotherapy), were considered eligible for this study (Figure 1).

To be included in the study, patients needed to have a recent (within 6 months) X-ray and an MRI or ultrasound of the affected shoulder. Other inclusion criteria were as follows:

- Consent to participate in the study.
- Severe overall pain and night pain: Numerical Rating Scale (NRS) for pain [8] above or equal to 60/100; the scale was modified from 0-10 to 0-100 (0 = no pain 100 = unbearable pain).
- No significant improvement after at least 2 courses of physiotherapy (including direct assistance from a physical therapist with specific exercises, manual therapy, and physical agents in order to reduce inflammation and pain).
- Clinical signs of subacromial tendinobursitis, with or without signs of rotator cuff tear, defined as positive Neer, Yocum, and Hawkins tests [9–15]. Pain experienced during the execution of these tests needed to reproduce the type of pain that patients complained the most.
- No indication for surgery due to the age, concomitant comorbidities, or patient's refusal to undergo surgery.
- Well-controlled type 2 diabetes, defined as follows [16]:
  - Fasting and preprandial blood glucose levels in the range of 90-150mg/dL.
  - Hb1Ac <64mmol/mol (corresponding to 8%) measured within 6 months.
- A device for self-measurement of blood sugar levels at home.

Exclusion criteria were as follows:

- Corticosteroid treatment in the past 3 months.
- Complaints of shoulder stiffness more than shoulder pain.
- Symptomatic glenohumeral arthritis defined as shoulder stiffness plus moderate radiographic signs of arthritis (grade >2 according to Hamada classification [17] and grade >1 according to Samilson-Prieto classification for eccentric and concentric arthritis, respectively [18]).
- Shoulder trauma within 3 months of inclusion evaluation for in this study.
- High blood pressure values (systolic blood pressure >140mmHg, diastolic blood pressure >80mmHg [16]).
- Glaucoma [19].

After an initial screening, patients were included in a prestudy phase and baseline data were recorded, including type of antidiabetes medications, range of motion (ROM), overall and night pain (NRS), Constant Score (CS) [20, 21], Oxford Shoulder Score (OSS) [22, 23], and Subjective Shoulder Value (SSV) [24].

Moreover, we asked the patients to measure their glycemia 4 times a day (before breakfast [fasting glycemia], 2 hours after lunch, before dinner [preprandial], and 2 hours after dinner) for one week after the first examination. These data were used as control values and to confirm that patients had well-controlled diabetes. In this prestudy phase patients did not receive any injection, but only NSAIDs and painkillers were prescribed. A measurement of Hb1Ac for the patients that did not have one recent blood test (within 6 months) was also prescribed.

A subsequent examination was scheduled after one week (Figure 1) when the principal investigator focused on the average fasting glycemia the week before the consultation and on the Hb1Ac. Hence, patients were either kept in the study or excluded if the diabetes was not under control. Patients with a mean fasting blood glucose of 90-150mg/dl and Hb1Ac<8% were included in the study. Patients were excluded if shoulder pain subsided after NSAIDs and painkillers previously prescribed

After discussing the risks and benefits of the therapy, a subacromial injection of Methylprednisolone Acetate 40 mg (Depo-Medrol©) and 2 ml of Lidocaine 2% was performed the morning of day 7. The same expert shoulder surgeon performed the injections using the posterior approach to the shoulder [25, 26]. The portal was located 1 cm inferior to the posterolateral border of the acromion. The needle was directed slightly superiorly, anteriorly, and medial toward the subacromial space. An ultrasound monitoring of the injection was not performed. Immediate, partial pain relief due to the Lidocaine indirectly confirmed the correct site of injection.

Patients were asked to continue the same regimen of physical activity, rehabilitation, and antidiabetes medications. Patients were informed of the probable increasing of blood glucose levels and were asked to cut down on saturated fats, salt, and sugars. Severe hyperglycemia was defined as a single value of fasting/preprandial glucose blood levels higher than 250 mg/dL or postprandial glucose blood levels higher than 300 mg/dL. Patients were also asked to report any abnormal symptoms or signs (systolic and diastolic blood pressure, respectively, over 140 mmHg and 80 mmHg [16], nausea, erythema, itching, or vomiting). Patients were requested to stay in the clinic for 30 minutes after the injection to diagnose any immediate adverse reaction. After the injection, patients were requested again to measure and record their blood sugar levels 4 times a day for the following 7 days. After 30 days, a follow-up examination was scheduled in our outpatient clinic.

At follow-up, outcomes were evaluated by using the CS, OSS, Subjective Outcome Determination (SOD) score [27, 28], and SSV. The questionnaires were filled out in the clinic. If patients reported insufficient pain relief or the recurrence of shoulder pain, a second subacromial injection was proposed. In this situation, the patient was not excluded from the study but we asked the patient to again measure their blood sugar levels 4 times a day.

Six months after the injection, a telephone interview was performed to assess pain, OSS, and SOD score.

The study was approved by the Institutional Review Board of our Hospital (Prot. CS/488) and have therefore been performed in accordance with the ethical standards laid down in the 1964 Declaration of Helsinki. All persons gave their informed consent prior to their inclusion in the study.

### 2.1. Statistical Analysis

A power analysis was performed. A significant reduction of the overall pain was considered as principal outcome. Eighteen patients were required for an expected reduction of 30 points of pain, with *α*: 0.05 and *β*: 0.2. The data collected were analyzed with Student's t-test, Fisher test, and the McNemar test for paired proportions. The blood glucose levels at each different time point were compared to the baseline levels (i.e., 7 days prior to the injection) using the Student's t-test. All the collected variables had a normal distribution except the SOD score. The D'Agostino-Pearson test for normal distribution rejected a normal distribution for the SOD score. The median of SOD score was therefore reported.

## 3. Results

Five out of 30 patients were excluded in the prestudy phase, since their diabetes was not well controlled. An additional 5 patients were excluded because they did not adequately record their glycemia after the injection (2 patients) or did not come back for the follow-up examination (3 patients). The 3 patients lost to follow-up were contacted by phone and they stated a significant improvement after injection. Twenty patients were ultimately included in the study. One patient was subsequently lost to follow-up at 6 months during the telephone interview phase. This patient was not excluded since the majority of the data important for the study were available for the analysis.

The average age was 71 years (63-83), 12 patients were female, 7 were managing their diabetes with insulin therapy, and 13 were doing so with oral diabetes medications. The average baseline CS was 43±12, the OSS 28±8, and the NRS for pain 76±11. All the patients had positive tests for subacromial tendinobursitis. Four patients had a concomitant adhesive capsulitis. The imaging studies showed a cuff tear involving the supraspinatus in 12 patients, a massive cuff tear involving the infraspinatus and supraspinatus in 4 patients. Four patients had mild signs of eccentric osteoarthritis (Hamada [17] stages 1 and 2).

### 3.1. Efficacy

**Figure 2:**
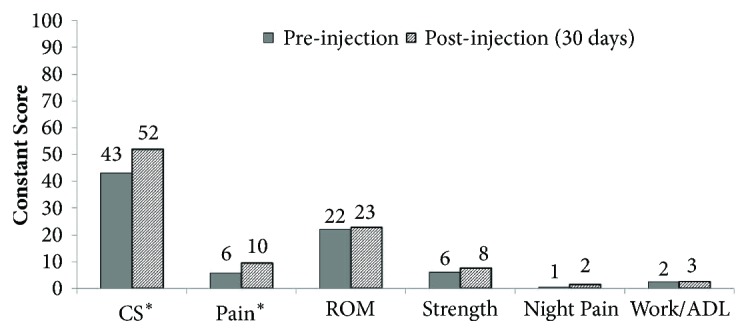
Preinjection and postinjection Constant Score (CS). The figure shows the improvement of the CS after subacromial corticosteroid injections. The improvement is significant (*∗*= p<0.05) for the overall CS and for the pain item. Details regarding night pain are reported in Figure 3.

**Figure 3:**
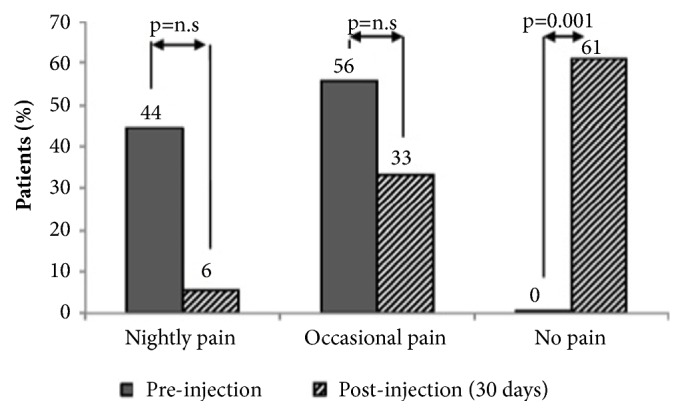
Preinjection and postinjection night pain. A significant improvement in night pain was recorded in the study. The rate of patients who experienced nightly pain decreased after the injection. Simultaneously, the rate of patients that did not have night pain increased to 61% after the injection.

At 30 days of follow-up a statistically significant improvement of the CS was reported, from 43±12 to 52±13 (p=0.008). The improvement in CS was more relevant considering the pain component of the score (Figures 2 and 3).

The variables, strength, ROM, and Work/ADL, did not improved significantly after the injection.

**Figure 4:**
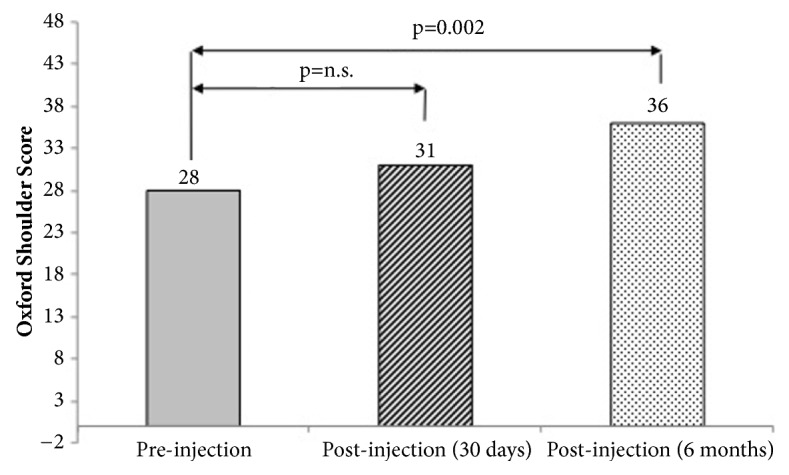
Oxford Shoulder Score (OSS) before and after injection. The improvement of the OSS was significant only after 6 months.

An improvement was also measured using the OSS but was only significant at 6 months (Figure 4).

The overall pain improved from 76±11 to 44±22 (p<0.001) at 30-day follow-up and to 38±30 at 6-month follow-up (p<0.001). However, the improvement in overall pain between 30 days and 6 months was not significant (p=n.s.). Nightly pain was reduced from 78±10 to 35±19 (p<0.001) at 30 days and to 31±25 (p<0.001) at 6-month follow-up. The improvement in nightly pain levels between 30 days and 6 months was not significant (p=n.s.).

The SSV preinjection was 49±14; it improved to 64±19 thirty days after injection (p=0.001) and to 68±25 (p<0.001) at 6-month follow-up. Again, the improvement between 30 days to 6 months was not statistically significant (p=n.s.).

One month after injection, the clinical improvement was also shown by a median SOD score of 6 points (= “Greatly Improved”). The median SOD value at 6 months remained 6 points.

During the study period, 5 patients asked for a second subacromial injection.

### 3.2. Safety

**Figure 5:**
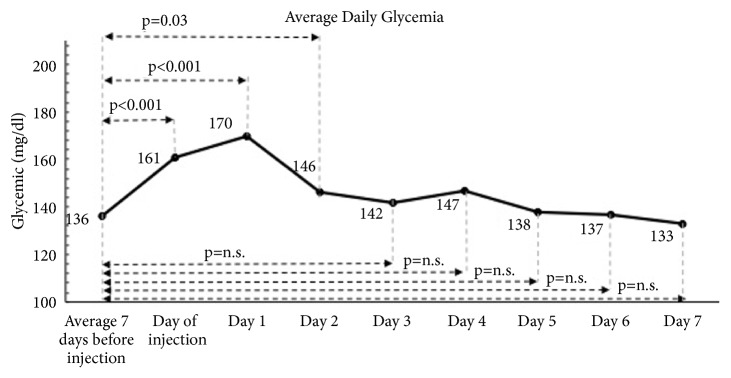
Increase of blood glucose levels after subacromial corticosteroid injections. The average daily values were 136 mg/dL (SD=28mg/dL) before the injection; they increased to 161 mg/dL (SD=25) the day of the injection (p<0.001) and to 170 mg/dL (SD=31) the day after the injection (p<0.001).

The average daily blood glucose levels are reported in Figure 5. Blood glucose levels were not statistically different after 3 days after injection. After 5 days blood glucose levels were normal.

**Figure 6:**
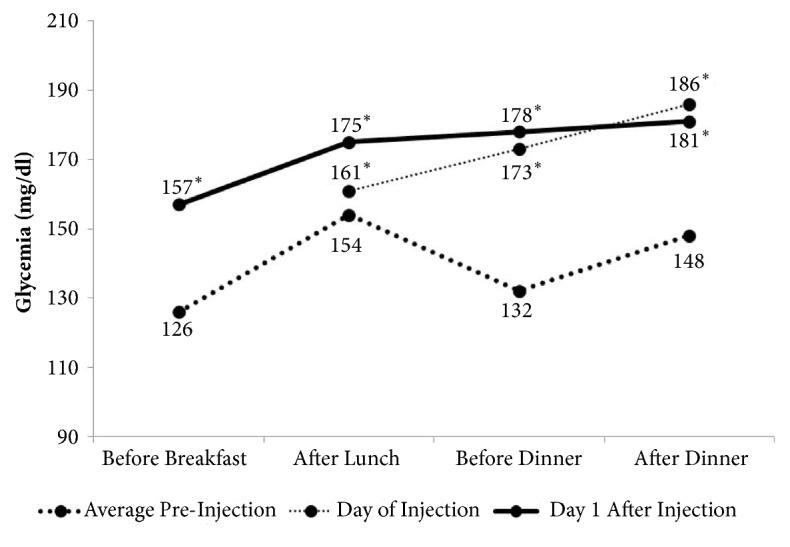
Daily glycemia the day and the days after the injection. One hour after lunch, the day of the injection (which was performed in the morning), the average glycemia was not different from the control values (161mg/dL, SD= 46.4; p=0.8). Glycemia increased significantly compared to the control values, before dinner (173mg/dL, SD=52.3 versus 132 mg/dL, SD=18.9; p=0.003) and after dinner (186mg/dL SD=54.2 versus 148 mg/dL, SD=42.2; p<0.001). The day after the injection, all glycemic values were significantly different from the control values. Before breakfast glycemia was 157 mg/dL SD= 50.7 (p=0.02), 2 hours after lunch 175 mg/dL SD=69.8 (p=0.03), before dinner 178 mg/dL SD=63.9 (p<0.001), and after dinner 181 mg/dL SD=48.5 (p=0.01).

Figure 6 shows that the details of the blood glucose levels increase the day of and the day after the injection. An increase of blood sugar levels was observed the day of and the day after the subacromial injection.

Five patients (25%) had severe hyperglycemia: 2 patients had one episode of fasting/preprandial glycemia >250mg/dL the day after the injection, 2 patients had a postprandial glycemia >300mg/dL (330 and 308 mg/dl), 1 patient had one episode of fasting glycemia of 265 mg/dL followed by a postprandial glycemia of 304 mg/dL. All the episodes of severe hyperglycemia were recorded the day and the day after the injection. Three out of these 5 patients were managing their diabetes with oral medications.

A posthoc analysis of the patients that experienced severe hyperglycemia revealed that they had an average preinjection fasting glycemia worse than the patients who were not affected by this complication (137mg/dl, range 113 to 150 versus 122 mg/dl, range 84 to 150).

One patient reported insomnia the night of the injection not related to shoulder pain. No other complications were reported.

## 4. Discussion

The aim of this prospective study was to report the short-term benefits and the effects on glucose blood levels of a subacromial injection with corticosteroids in type 2 diabetic patients affected by severe shoulder pain, not responsive to conservative treatment and not suitable for surgery. The most important finding is that the injection significantly reduced the overall pain and the night pain and most of the patients had a perception of a greatly improved shoulder at 1- and 6-month follow-up.

These data were consistent with previous reports. Yu [29] showed, in a prospective clinical study conducted on 238 shoulders, a significant improvement of the quality of life and of the ROM in 91% of cases 1 month after the injection with 1 ml of Xylocaine 2% and 1 ml of Betamethasone. One year later, the improvement was still significant in 88% of the treated patients. Akgun [30] highlighted a substantial improvement of shoulder symptoms, by using the CS 1 month after injection, due moreover to the reduction of pain at night.

In contrast to what has been reported previously, we did not observe a significant improvement in ROM and function after subacromial injection. The reason could be a difference in the patient population included in this study. We included older diabetic patients affected by shoulder pain with multiple etiologies including rotator cuff tears and mild osteoarthritis. Moreover, despite the fact that a clear diagnosis of adhesive capsulitis was present in only 4 cases, we cannot rule out the possibility that some of the patients had some minor forms of adhesive capsulitis secondary to diabetes [31, 32]. The infiltration could have improved the pain related to subacromial tendinobursitis and long head of the biceps inflammation but perhaps was unable to improve the ROM mostly due to an inflamed and tight capsule secondary to adhesive capsulitis or osteoarthritis.

The second finding was the effect of subacromial injections on blood glucose levels in type 2 diabetic patients. Overall, the infiltration increased the average glycemia for three days, with a peak the day after the injection. No hyperglycemia-related symptoms were recorded. Although these data can be considered generally a good result, they must be interpreted with extreme caution. Twenty-five percent of the patients had a transitory severe hyperglycemia. These data are in contrast with the study of Habib [6] who showed that there is no significant change in blood glucose levels after injecting 35 mg of Methylprednisolone Acetate at the shoulder joint. The reason for this difference is unknown. One potential explanation is that the effect on blood glucose levels of an intra-articular injection of Methylprednisolone Acetate is different from a subacromial injection. The absorption of the corticosteroids could be higher and faster in the inflamed subacromial bursa. Other reasons could be due to, in our study, 40 mg instead of 35 mg of Methylprednisolone Acetate being injected and that the corticosteroid was associated with Lidocaine.

Interestingly, in our series the patients that had severe hyperglycemia had had a suboptimal fasting glycemia before the injection. This data could imply that a better control of diabetes should prevent postinjection hyperglycemia and improve the safety of the treatment with corticosteroids.

The comparison of our results with other studies [1–3, 5, 6, 33] revealed that the effect of corticosteroid injections in other joints is also a variable and in some cases can significantly increase the blood glucose levels. Habib [1] reported a short glycemia increase only the first day after Celestone Chronodose in 6 knees of patients affected by type 2 diabetes. In 30 diabetic patients who underwent an injection with Triamcinolone Acetonide and Triamcinolone Esacetonide, a highly variable increase in glycemic values was reported (always below 300 mg/dL). Zufferey [5] reported no change in blood glucose levels among patients who received an epidural injection with 80 mg of Methylprednisolone Acetate, while patients who underwent an injection in their knees or shoulders with the same drug recorded an increase of glycemia, with a high individual variability.

One option for the treatment of persistent shoulder pain in diabetic patients is the injection with hyaluronic acid. However, the effect of this injection in diabetic patients has never been proven effective. Three patients in our study cohort have been injected with hyaluronic acid before being included in our study. None of them had reported a significant improvement.

Penning [34] compared subacromial Triamcinolone Acetonide, hyaluronic acid, and placebo for shoulder pain: he observed a booster effect in pain reduction after repeated Triamcinolone Acetonide injections. The hyaluronic acid and placebo group showed only slight improvements in pain reduction.

This study has some limitations. The injections were performed without use of ultrasound guidance. A study, however, showed that there is little evidence for the need of ultrasound guided needle placement [35]. Our patients reported an immediate slight to moderate benefit from the injection, indirect proof that the injection, that contained Lidocaine, was performed correctly. Another limitation is that we included diabetic patients with resistant shoulder pain, regardless the type of diagnosis. The main goal of this study was to test the short-term benefit of corticosteroid injections on pain. Shoulder pain, in old patients, has a multifactorial etiology. For this reason, we preferred to include all the patients with pain and not only those affected by a specific pathology. Future studies should address the risk-benefit ratio of corticosteroid injections in specific pathologies.

## 5. Conclusions

Subacromial injection with 40 mg of Methylprednisolone Acetate is effective as a short-term treatment in type 2 diabetic patients affected by shoulder pain. Patients must be informed about the possibility of transient hyperglycemia for the 3 days after injection and that 20% could have a transient severe hyperglycemia, especially the day of and the day after the injection and they must be closely followed up.

## Data Availability

The data that support the findings of this study are available on request from the corresponding author (Davide Blonna).
